# Supplementary material for: Changes in Cat Facial Morphology Are Related to Interaction with Humans
Source: Animals (Basel). 2022 Dec 10;12(24):3493. doi: 10.3390/ani12243493 (PMC9774281; doi:10.3390/ani12243493)
Supplement: Supplementary file 1 [file animals-12-03493-s001.zip › Supplementary_Materials_1027.pdf]

Supplementary Materials

Table S1. List of owned domestic purebreds.

Breed	n
Abyssinian	50
American Bobtail	50
American Curl	50
American Shorthair	50
American Wirehair	33
Australian Mist	20
Balinese	25
Bengal	50
Birman	50
Bombay	50
British Shorthair	50
Burmese	50
Burmilla	50
Chartreux	50
Chausie	20
Colorpoint Shorthair	20
Cornish Rex	50
Cymric	22
DevonRex	50
Donskoy	23
Egyptian Mau	50
European Shorthair	20
Exotic	50
Havana Brown	50
Himalayan	27
Japanese Bobtail	50
Khao Manee	50
Korat	50
Kurilian Bobtail	34
LaPerm	32
Lykoi	22
Maine Coon	50
Manx	50
Minuet	31
Munchkin	34
Nebelung	26
Norwegian Forestcat	50
Ocicat	50
Oriental	50
Persian	50
Peterbald	32
Pixiebob	27
Raga Muffin	29
Ragdoll	50
Russian Blue	50
Savannah	22

Scottish Fold	50
Selkirk Rex	50
Siamese	50
Siberian	50
Singapura	50
Snowshoe	22
Sokoke	20
Somali	50
Sphynx	50
Thai	20
Tonkinese	50
Toyger	23
Turkish Angora	50
Turkish Van	50

Table S2. List of Fe-BARQ sections.

Factor	Number of items
General activity/Playfulness	14
Sociability with people	7
Directed vocalization	4
Purring	2
Attention-seeking	2
Sociability with cats	3
Stranger-directed aggression	3
Owner-directed aggression/Touch sensitivity	4
Resistance to restraint	4
Familiar cat aggression	4
Dog aggression	5
Fear of unfamiliar dogs/cats	2
Fear of novelty	2
Separation-related behavior	6
Trainability	3
Predatory behavior	3
Prey interest	2
Sleeping location preferences	3
Excessive/compulsive self-grooming	3
Other compulsive behaviors	3
Inappropriate elimination	2
Elimination preferences	2

Table S3. Attributes of the survey participants (whether they own cats, like cats, or like dogs).

	All	Who like cats	Who like cats as much as dogs	Who like dogs
Males with experience in cat ownership	44	28	9	7
Males with no experience in cat ownership	43	14	7	22
Females with experience in cat ownership	144	115	24	5
Females with no experience in cat ownership	121	52	26	43
